# Dual Application of Waste Grape Skin for Photosensitizers and Counter Electrodes of Dye-Sensitized Solar Cells

**DOI:** 10.3390/nano12030563

**Published:** 2022-02-07

**Authors:** Yuan Yuan, Caichao Wan

**Affiliations:** 1 College of Art and Design, Hunan Vocational College of Science and Technology (Hunan Porcelain College), Changsha 410004, China; yyuan202202@163.com; 2 College of Materials Science and Engineering, Central South University of Forestry and Technology, Changsha 410004, China

**Keywords:** grape skin, carbon, photosensitizer, counter electrodes, dye-sensitized solar cells

## Abstract

Dye-sensitized solar cells (DSSCs), a powerful system to convert solar energy into electrical energy, suffer from the high cost of the Pt counter electrode and photosensitizer. In this study, the dual application of waste grape skin is realized by employing the grape skin and its extract as the carbon source of the carbon-based counter electrode and photosensitizer, respectively. The ultraviolet–visible absorption and Fourier transform infrared spectroscopy verify the strong binding between the dye molecules (anthocyanins) in the extract and the TiO_2_ nanostructure on the photoanode, contributing to a high open-circuit voltage (*V*_OC_) value of 0.48 V for the assembled DSSC device. Moreover, the waste grape skin was subjected to pyrolysis and KOH activation and the resultant KOH-activated grape skin-derived carbon (KA-GSDC) possesses a large surface area (620.79 m^2^ g^−1^) and hierarchical porous structure, leading to a high short circuit current density (*J*_SC_) value of 1.52 mA cm^−^^2^. Additionally, the electrochemical impedance spectroscopy reveals the efficient electron transfer between the electrocatalyst and the redox couples and the slow recombination of electrolytic cations and the photo-induced electrons in the conduction band of TiO_2_. These merits endow the DSSC with a high photovoltaic efficiency of 0.48%, which is 33% higher than that of a common Pt-based DSSC (0.36%). The efficiency is also competitive, compared with some congeneric DSSCs based on other natural dyes and Pt counter electrode. The result confirms the feasibility of achieving the high-value application of waste grape skin in DSSCs.

## 1. Introduction

**Figure 1:**
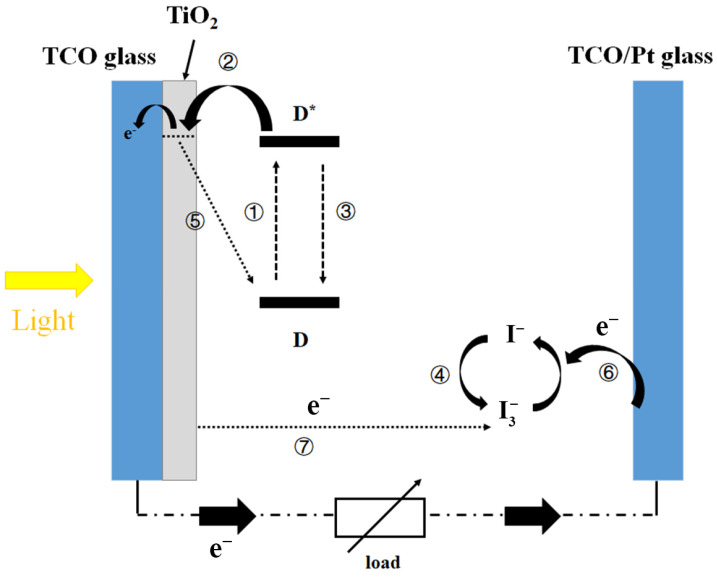
Working principle of a DSCC device composed of a TiO_2_-coated TCO photo-anode, a TCO/Pt glass counter electrode, an iodine-based electrolyte, and a photosensitizer (dye molecules).

The energy crisis and environmental problems caused by the massive consumption of fossil fuels have greatly stimulated people’s enthusiasm for clean sustainable energy resources. Among them, solar energy is considered to be the most promising renewable energy [1,2,3]. According to statistics by the Intergovernmental Panel on Climate Change (IPCC), the photon flux received by the Earth from the Sun reaches up to 120 KTW of energy, far exceeding the average output of other renewable energy sources such as winder energy (2–4 TW), hydroelectric energy (~0.5 TW), tide energy (<2 TW), and geothermal energy (12 TW) [4,5]. Thus, converting adequate and nature-friendly solar radiation into energy is undoubtedly a clear, logical, and economic goal [6,7,8]. Compared to traditional silicon solar cells, dye-sensitized solar cells (DSSCs) have attracted much attention due to their low cost and high efficiency. DSSCs based on wide-bandgap metal oxide semiconductors and redox couple electrolytes are considered a clean and cost-effective way to convert solar energy into electrical energy [9,10,11]. A typical DSSC consists of photo-anodes (such as transparent conductive oxides (TCOs) coated with nano-TiO_2_), counter electrodes, dye molecules that absorb light to produce photoelectrons, and electrolytes containing redox pairs. The main working principle of DSSCs can be described below [12,13]: (1) the dye molecule located above the edge of the conduction band (CB) of semiconductor nanoparticles (such as TiO_2_) absorbs photons (*hν*) and transfers to the electron excited state D* (Equation (1)) and then injects an electron into the CB (Equation (2)); (2) the deactivation reaction (Equation (3)) is the relaxation of the excited state, which competes with the electron injection. Equation (5) (back electron transfer) and Equation (7) (redox pair electron capture of CB) show two charge recombination processes, which are key reasons that hinder electron collection efficiency by light injection; (3) the two processes compete with the oxidation of iodide (Equation (4)) and reduce the cell’s performance. The reduction in iodine is accomplished by electrons injected through an external circuit (Equation (6)). Figure 1 illustrates the workflow of DSCCs:

(1) $$D+h\nu \to D^{*}$$

(2) $$D^{*}+{\mathrm{TiO}}_{2}\to D^{+}+e_{cb}^{-}({\mathrm{TiO}}_{2})$$

(3) $$D^{*}\to D$$

(4) $$2D^{+}+3I^{-}\to 2D+I_{3}^{-}$$

(5) $$D^{+}+e_{cb}^{-}({\mathrm{TiO}}_{2})\to D+{\mathrm{TiO}}_{2}$$

(6) $$I_{3}^{-}+2e^{-}(\mathrm{catalyst})\to 3I^{-}$$

(7) $$I_{3}^{-}+2e_{cb}^{-}({\mathrm{TiO}}_{2})\to 3I^{-}+{\mathrm{TiO}}_{2}$$

To improve the efficiency and stability of DSSCs and reduce their costs, intensive studies have been conducted to improve the main components of DSSCs, including photoanodes, counter electrodes, electrolytes, and a photosensitizer [8,14,15,16]. Therein, the photosensitizer is one of the essential parts of DSSCs, and its primary function is to absorb sunlight and then convert the absorbed sunlight into electricity [17]. Therefore, people have dedicated a lot of energy to designing dye molecules that can absorb the solar spectrum better to obtain higher output performance. At present, the commonly used photosensitizer is the ruthenium complex, which is mainly due to its wide visible light absorption range, long excited-state lifetime, and basically matching the excitation energy of the TiO_2_ CB [18,19]. However, the use of expensive ruthenium compounds increases the production cost of DSSCs and leads to a relatively large environmental load. Compared with synthetic dyes, natural dyes possess advantageous features such as their easy availability, mass availability, no need for purification, and no pollution, thus significantly reducing the cost of equipment. In natural pigments, there are four kinds of compounds that are mainly used as photosensitizers of DSSCs: chlorophylls [20,21]; flavonoids [22,23]; carotenoids [24]; and anthocyanins [25]. In addition, the electrocatalyst in the paired electrodes plays a vital role in determining the overall photovoltaic efficiency of DSSCs since the correct selection of electrocatalysts helps improve the I^−^/I_3_^−^ reduction reaction and prevent the recombination of oxide cations (cationic form of photosensitizers) with electrons in the semiconductor CB [6]. Pt is a common electrocatalyst in DSSCs because of its high electrocatalytic activity, chemical stability, and exchange current density [26]. However, Pt is an expensive metal which defeats the whole purpose of achieving low-cost DSSCs. In addition, Pt is easy corroded in traditional iodine electrolytes. By comparison, porous carbon is a promising candidate to substitute the Pt electrocatalyst. Porous carbon has a high specific surface area, adjustable pore size, large pore volume, and good interconnected pore structure, which are conducive to the free transfer of ions, and the reduction in charge transfer resistance [27].

**Figure 2:**
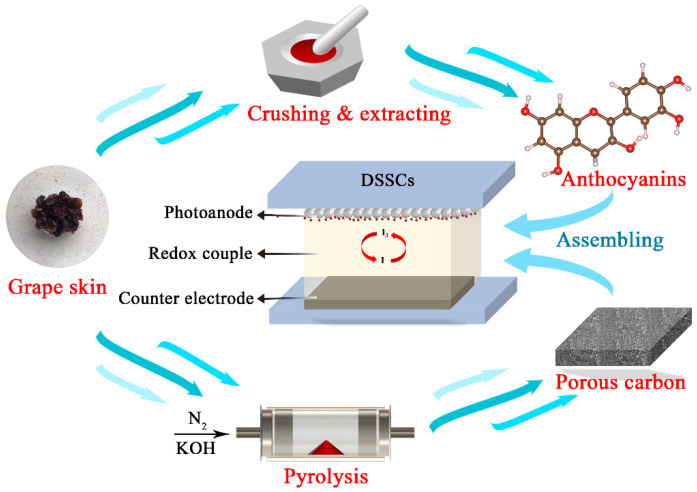
Schematic diagram for the dual application of grape skins in DSSCs.

Summer black grape has a thick pericarp with a uniform black-blue color and is rich in anthocyanins [28]. However, the grape skins are often discarded as waste, which undoubtedly causes a waste of biomass resources and even environmental pollution. In this work, for the sake of reducing the cost of DSSCs and improving the additional value of grape skins, the dual application of grape skins in DSSCs was achieved by utilizing grape skins as the feedstock of photosensitizer and the precursor of carbon electrocatalysts. The fabrication process is illustrated in Figure 2. This high performance-to-price ratio of DSSC acquires a favorable photoelectric conversion efficiency of 0.48%, slightly higher than that of the Pt electrocatalyst-based DSSC (0.36%) and competitive with numerous congeneric products. Therefore, these results demonstrate the high application potential of grape skins in DSSCs.

## 2. Materials and Methods

### 2.1. Materials

High-performance iodine solution electrolyte (LNT-DE02), Suryn film (60 μm), and fluorine-doped tin oxide (FTO) conducting glass were purchased from Liaoning Libra Technology Co., Ltd. (Shenyang, China). Anhydrous ethanol, TiCl_4_ and KOH were obtained from Sinopharm Chemical Reagent Co., Ltd. (Shanghai, China). TiO_2_ slurry (WH20, anatase) was supplied from Shanghai Macklin Biochemical Co., Ltd. (Shanghai, China). All reagents can be used directly without any further purification.

### 2.2. Preparation of Photoanode Using the Extract of Grape Skin as Photosensitizer

#### 2.2.1. Extraction of Natural Dye from Grape Skin

Clean and fresh grape skins were washed with distilled water and then ground in a mortar. A proper amount of ethanol was added to extract the natural pigment from the grape skin. Thereafter, the crude extract was transferred to a centrifuge and centrifuged at 8000 r min^−1^ for 30 s. The supernatant of the centrifugal product was employed as the photosensitizer of the photoanode, and the precipitate was dried and used as the feedstock of the counter electrode.

#### 2.2.2. Preparation of TiO_2_ Electron Transport Layer

The TiO_2_ electron transport layer consists of two main layers, i.e., the dense underlayer and superficial layer for dye adsorption. For the preparation of the dense underlayer, FTO was successively rinsed with toluene, acetone, ethanol, and distilled water by ultrasonic oscillation for 30 min and then air-dried. The clean FTO was vertically placed in a beaker, and then the TiCl_4_ aqueous solution with a concentration of 40 mM was added to the beaker until the liquid completely reached the top of FTO. After that, the FTO was removed and put in an oven at 70 °C for 30 min, promoting the hydrolysis of TiCl_4_ and the generation of TiO_2_. The above process was repeated once to ensure the formation of a thin and dense TiO_2_ bottom layer. Finally, the organic matter on the surface was wholly removed by annealing at 450 °C for 30 min.

For the preparation of the superficial layer for dye adsorption, the TiO_2_ bottom layer-coated FTO was further covered with the WH20 TiO_2_ slurry through a screen-printing method. The thickness was controlled by adjusting the gap distance between the screen-printing mesh and the FTO by using the two bolts which controlled the height between the substrate suction base and the screen-printing mesh. The thickness was approximately 12 μm. After screen-printing, the TiO_2_ layer was sintered again at 450 °C for 30 min.

#### 2.2.3. Sensitization Process

The sensitization process was carried out by dipping the sintered TiO_2_ film-coated FTO in the natural dye extracted from the grape skin for 24 h. Thereafter, the floating color was washed off with ethanol and then the clean dye-sensitized TiO_2_ film-coated FTO was air-dried. The resulting photoanode was successfully prepared.

### 2.3. Preparation of Counter Electrode Using Grape Skin Residue as Carbon Resource

#### 2.3.1. Preparation of Grape Skin-Derived Carbon (GSDC)

After being washed and dried, the clean and dried grape skin residue was transferred to a tube furnace. Under the protection of N_2_, the grape skin residue was heated to 500 °C at a rate of 5 °C min^−1^ and kept for 1 h, before continued to be heated to 1000 °C at a rate of 5 °C min^−1^ and kept at the temperature for 2 h. Hereafter, the temperature is reduced to 500 °C at a cooling rate of 5 °C min^−1^ and finally naturally cooled to room temperature. The grape skin-derived carbon (GSDC) was obtained.

#### 2.3.2. KOH Activation of GSDC

One gram of GSDC and four grams of KOH were mixed with 50 mL of deionized water and then a beaker containing the mixture was gently shaken in a constant-temperature oscillator for 1 h. After being dried at 105 °C, the mixture was calcined in a tubular furnace under the protection of argon. The temperature was increased from room temperature to 800 °C at 10 °C min^−1^ and was then kept at 800 °C for 5 h. After heating, the sample was naturally cooled to room temperature. The calcinated sample was washed with hydrochloric acid until pH = 7. Finally, the sample was dried at 60 °C and named KOH-activated GSDC (KA-GSDC).

#### 2.3.3. Assembly of Counter Electrode

A small amount of TiO_2_ slurry was evenly mixed with the KA-GSDC and a small amount of ethanol. A thin film was prepared on FTO by using a filming coating machine. The modified FTO was placed at room temperature for 1 h and then transferred to a tube furnace to undergo a calcination treatment at 450 °C for 30 min under nitrogen protection.

### 2.4. Fabrication of DSSCs

The photoanode and counter electrode were stacked and separated by a Suryn film to form a hollow sandwich structure. Then, a hot air gun was used to heat the sandwich structure for the sake of melting the Suryn film and achieving the bonding purpose. The electrolyte was injected into the gap between the photoanode and counter electrode with a syringe. The excess electrolyte was wiped off with dust-free paper and sealed with UV glue.

### 2.5. Characterizations

The absorbance of grape skin extract before and after TiO_2_ sensitization was studied by an ultraviolet–visible (UV–Vis) spectrophotometer (UV-2550, Shimadzu, Kyoto, Japan). The surface morphology and element composition were analyzed by scanning electron microscope (SEM, Sigma 300, Carl Zeiss, Oberkochen, Germany) and energy-dispersive X-ray (EDX) spectroscopy, respectively. Raman spectra were recorded on a Raman spectrometer (LabRAM HR Evolution, HORIBA Scientific, Paris, France) adopting an argon-ion laser (514 nm) as the excitation source. X-ray diffraction (XRD) patterns were recorded on a Bruker D8 Advance TXS XRD instrument with Cu K_α_ (target) radiation (*λ* = 1.5418 Å) at a scan rate (2*θ*) of 4° min^−1^ in the scan range of 10–80°. FTIR spectra were recorded on a FTIR spectrophotometer (Magna 560, Nicolet, Thermo Electron Corp, Madison, WI, USA) in the range of 400–4000 cm^−1^ with a resolution of 4 cm^−1^. 1D ^1^H nuclear magnetic resonance (NMR) spectra were recorded at 300 K on a NMR spectrometer (WNMR-I 400MHz, Q. One Instruments Ltd., Wuhan, China) by using a 5 mm inverse probe and fitted with an autosampler. The pore features were studied by a specific surface area and pore size analyzer (AUTOSORB IQ, Quantachrome, Boynton Beach, FL, USA). Photocurrent density-applied voltage (I–V) and electrochemical impedance spectroscopy (EIS) tests of the DSSCs were obtained by using a home-built setup comprising a xenon lamp, an AM 1.5 light filter, and a CHI660D Electrochemical Analyzer (CHI instruments). The power of the filtered light was calibrated by a China National Institute of Metrology certified silicon reference cell to 100 mW cm^−2^. The spectra of the incident photo to electron conversion efficiency (IPCE) were measured for the cells sensitized with the grape skin extract with the help of a solar cell QE/IPCE measurement system (CEL-QPCE2050, China Education Au-light Technology Co., Ltd., Beijing, China).

## 3. Results and Discussion

### 3.1. UV–Vis Absorption, FTIR, and NMR Spectroscopy of the Grape Skin Extract

**Figure 3:**
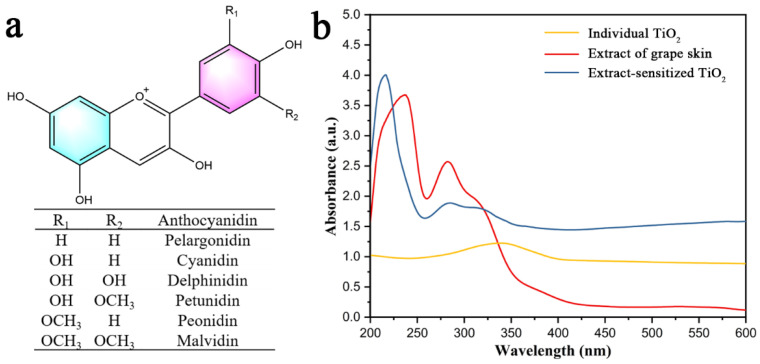
(**a**) Schematic diagram of the molecular structure of anthocyanins (the typical types of R_1_ and R_2_ are available in the table below); (**b**) UV–Vis absorption spectra of the individual TiO_2_ and the extract of grape skin before and after being adsorbed onto the TiO_2_ surface (solvent: anhydrous ethanol; concentration of the grape skin extract: ~0.72 mM).

Anthocyanins, a class of representative dye molecules for the photosensitizer of DSSCs, are rich in the extract of grape skin [28]. The molecular structure of anthocyanins is illustrated in Figure 3a. As a result, the extract of grape skin is a promising natural resource to develop an eco-friendly and inexpensive photosensitizer. UV–Vis absorption spectroscopy analysis was conducted to investigate the extract of the grape skin before and after being adsorbed onto the TiO_2_ surface. As shown in Figure 3b, the absorption for the individual TiO_2_ at approximately 350 nm is the characteristic of TiO_2_. By comparison, after the adsorption of the extract of grape skin by the TiO_2_, there are two clear absorption peaks in the range of 200−380 nm in the UV–Vis absorption spectrum of the extract-sensitized TiO_2_, which is similar to that of the extract of grape skin. The band centered at 240 nm was a feature of the carbinol base B structure of anthocyanins [29], and the band centered at 285 nm was associated with the aromatic group of anthocyanins [30]. For the extract of grape skin after being adsorbed onto the TiO_2_ surface, the band related to the carbinol base B structure shifts 24 nm to the lower wavelength. This blue shift phenomenon is because the dyes have a strong tendency to aggregate at the solid/liquid interface because the strong attractive forces between molecules and the H-aggregates (parallel orientation) will cause blue shift [31].

**Figure 4:**
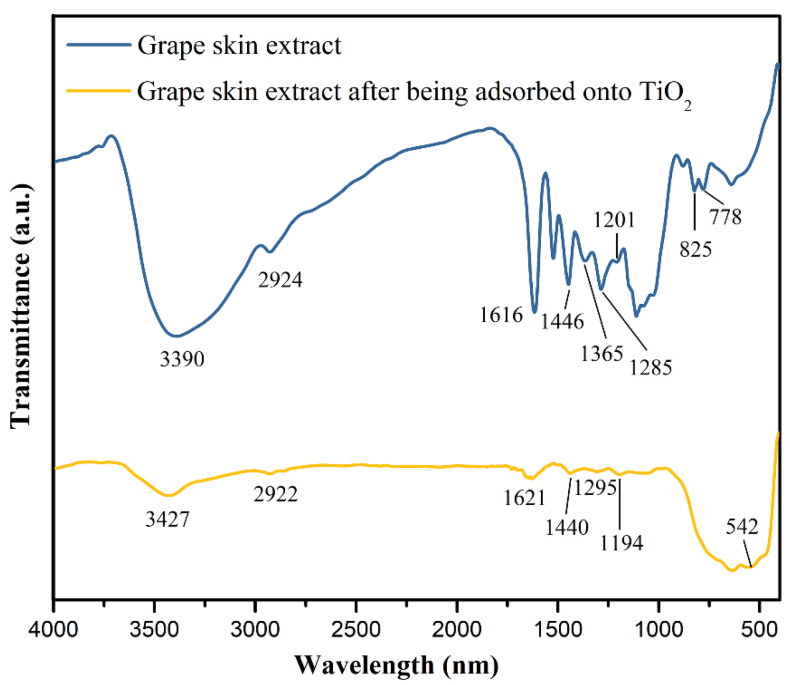
FTIR spectra obtained for the extract of grape skin before and after being adsorbed onto the surface of TiO_2_.

The FTIR characterization is carried out in the range of 400–4000 cm^−1^ to verify the type of chemical bonds in the extract of the grape skin. Figure 4 presents the FTIR spectra obtained for the extract of grape skin before and after being adsorbed onto the surface of TiO_2_. For the FTIR spectrum of the grape skin extract, a broad band in the range of 3700–3000 cm^−1^ is the stretching vibration of O–H groups, and the band appearing at 2924 cm^−1^ is ascribed to the C–H stretching [32]. The band at 1616 cm^−1^ is assigned to the C=C stretching vibration of aromatic rings from anthocyanins, while the band at 1365 cm^−1^ denotes the O–H bending of phenol [33]. A typical band localized at 1446 cm^−1^ originates from the OH–CH_2_ bonds of the phenolic rings presented in the anthocyanins [34]. In addition, the bands at 1285 and 1201 cm^−1^ are attributed to the C–O stretching from both phenolic groups and disaccharides that confirm the anthocyanins’ structure, and both bands at 825 and 778 cm^−1^ correspond to the C–H bending in the aromatic rings [32]. For the FTIR spectrum of the grape skin extract after being adsorbed onto the surface of TiO_2_, some characteristic bands of anthocyanins can still be clearly identified. Moreover, the band at 542 cm^−1^ is attributed to the Ti–O–Ti bonds of TiO_2_ [35], indicating the successful integration between the grape skin extract and TiO_2_. In addition, by comparing the FTIR spectra for the extract of grape skin before and after being adsorbed onto the surface of TiO_2_, we can find that the broad band of O–H stretching shifts from 3390 cm^−1^ (the extract) to the higher wavenumber of 3427 cm^−1^ (the extract/TiO_2_). This shift is due to the complexation between the –OH groups in anthocyanins and the Ti in TiO_2_ [36,37], which decreases the number of hydrogen bonds between anthocyanin molecules. This complexation implies a higher ordering in anthocyanins, which also lowers the H-bonding among these groups, causing a decrease in broadening. The result indicates both physical and chemical binding between the grape skin extract and the TiO_2_, thus revealing the potential of the extract of grape skin as a DSSC photosensitizer.

**Figure 5:**
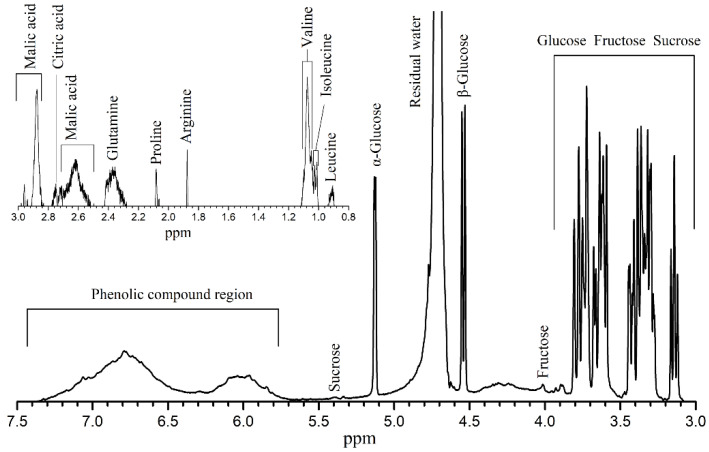
Representative ^1^H NMR spectrum for the grape skin extract (the sub-figure is the spectrum at low chemical shift).

The chemical components of the grape skin extract are determined by ^1^H NMR spectroscopy. Figure 5 presents a typical ^1^H NMR spectrum obtained at 500 MHz for grape skin extract. Signal assignment of the different extracts is identified after peak assignment using ^1^H NMR spectra from pure compounds associated with the comparison of published data [38,39]. In cases when further confirmation of the assignment is required, the extracts are spiked with appropriate standards to verify that the chemical shifts are identical. There are twelve main compounds that are identified in the ^1^H NMR spectrum of grape skin extract, as summarized in Table 1. The major resonances of the spectrum correspond to the phenolic compounds (anthocyanins), fructose, glucose and sucrose for the skin extract.

### 3.2. Microstructure, Elements, XRD Analysis, and Raman Spectroscopy of KA-GSDC

**Figure 6:**
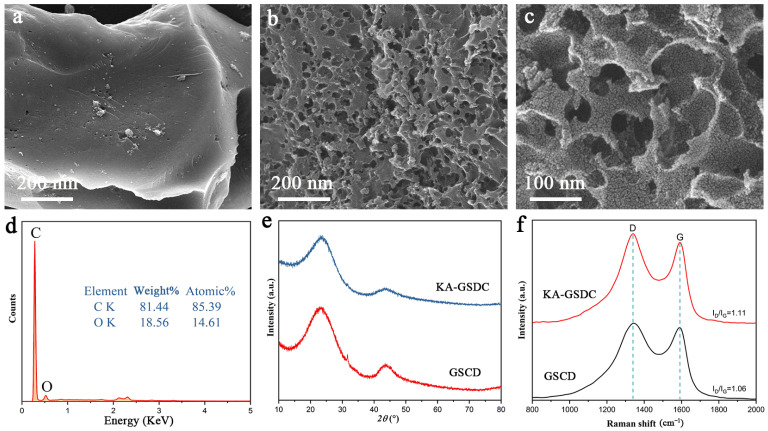
(**a**) SEM image of the GSDC; (**b**,**c**) SEM images and (**d**) EDX pattern of the KA-GSDC; (**e**) XRD patterns and (**f**) Raman spectra of the GSDC and KA-GSDC.

Because of the high carbon content, the grape skin residue is an ideal feedstock of carbon-based counter electrodes. Figure 6a,b provide the SEM images of the grape skin residue subjected to the pyrolysis (GSDC) and KOH activation (KA-GSDC). It can be seen that the surface of GSDC prepared by pyrolysis is smooth and presents few pores. After the activation by KOH, a hierarchical porous structure is formed for KA-GSDC (Figure 6c), indicating a higher potential surface area. An abundant pore structure with a large surface area is conducive to ion transfer and promotes the reduction reaction of electrolytes, which are of great significance to improve the cell’s performance [40]. To investigate the purity of the GSDC, an EDX analysis is carried out in Figure 6d, where there are primarily two signals including oxygen (18.56 wt%) and carbon (81.44 wt%) that are detected. The extremely low oxygen content suggests that the most oxygen-containing functional groups are removed.

Figure 6e shows the XRD patterns of GSDC and KA-GSDC. The two broad peaks centered at approximately 23.6° and 43.5° correspond to the (002) and (100) planes of amorphous carbon. Raman spectroscopy is a powerful tool to study the structure of carbon materials. Figure 6f presents the Raman spectra of GSDC and KA-GSDC. Two typical Raman bands, commonly known as D and G bands, appear very clearly at 1138 cm^−1^ and 1590 cm^−1^. The G-band is caused by the stretching of the C–C bond, which is common in all *sp*^2^ carbon systems. On the other hand, due to any disorder in the *sp*^2^ hybrid carbon system, the D band will appear and display an enhanced intensity, called the defect band. The *I*_D_/*I*_G_ intensity ratios of GSDC and KA-GSDC are 1.08 and 1.11, respectively. It is worth noting that a higher intensity ratio indicates a higher degree of defects, which is positively correlated with the fraction of mesopores [40]. Moreover, the previous study has demonstrated that the increased defect density contributes to the catalytic ability in DSSCs [41].

### 3.3. Pore Feature Analysis of KA-GSDC

**Figure 7:**
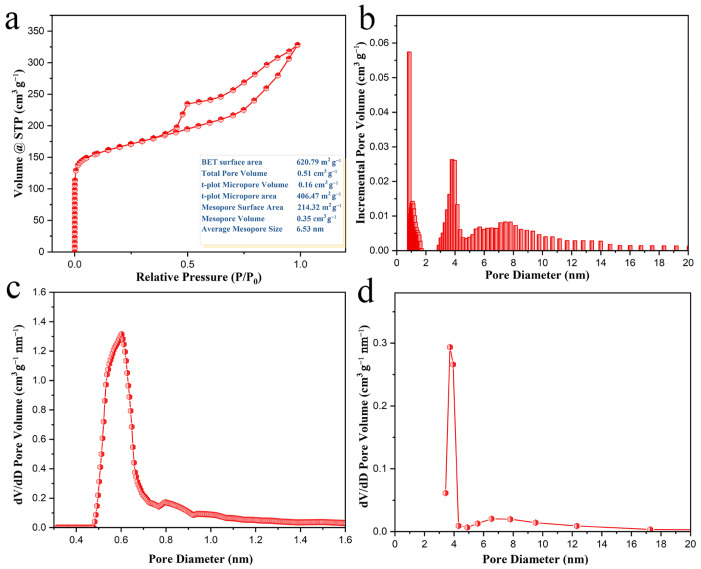
(**a**) N_2_ adsorption−desorption isotherms of KA-GSDC (the inset shows the pore characteristic data); (**b**) DFT pore volume distribution of KA-GSDC; (**c**) BJH mesopore size distribution of KA-GSDC; and (**d**) t-plot microporous pore size distribution of KA-GSDC.

To further study the pore feature of KA-GSDC, a N_2_ adsorption–desorption test is implemented to determine the specific surface area and pore diameter distribution. As displayed in Figure 7a, the adsorption isotherm belongs to a typical type IV isotherm with hysteresis, and the hysteresis loop is mainly in the middle and high-pressure region (*P*/*P*_0_ = 0.45−1) [42], which is the characteristic of mesopores (2−50 nm). Besides, the remarkably increased uptake in the low-pressure region reveals the presence of abundant micropores (<2 nm). The adsorption isotherm still does not reach a plateau near the *P*/*P*_0_ of 1.0, revealing the presence of macropores (>50 nm). Brunauer–Emmett–Teller (BET) and Barrett–Joyner–Halenda (BJH) analysis gives the specific surface area and pore volume of 620.79 m^2^ g^−1^ and 0.51 cm^3^ g^−1^, respectively. The BET surface area value of KA-GSDC is higher than that of many congeneric porous carbon products, for instance, cyanuric acid and melamine-derived activated carbon (305 m^2^ g^−1^) [43], porous carbon nanotube-glucose carbon foam (357 m^2^ g^−1^) [44], cellulose-derived carbon aerogel (113 m^2^ g^−1^) [45], and potassium humate-derived porous carbon (604 m^2^ g^−1^) [46].

The pore volume distribution diagram, calculated by the density functional theory (DFT) method, indicates that the porous structure of KA-GSDC consists of micropores and mesopores (Figure 7b). The result is further demonstrated by the t-plot microporous pore size distribution (Figure 7c) and the BJH mesoporous pore size distribution (Figure 7d). Clearly, the pores with sizes of approximately 0.6 nm and 4 nm primarily contribute to the pore volume. These hierarchical pores are beneficial for the electrocatalytic reaction in DSSCs. The large surface area of micropores (406.47 m^2^ g^−1^) provide abundant reaction active sites and the mesopores are capable of promoting the ion transport, ensuring higher electrocatalytic performance [47].

### 3.4. Assembly of DSSC Devices and Its J–V Characteristics

**Figure 8:**
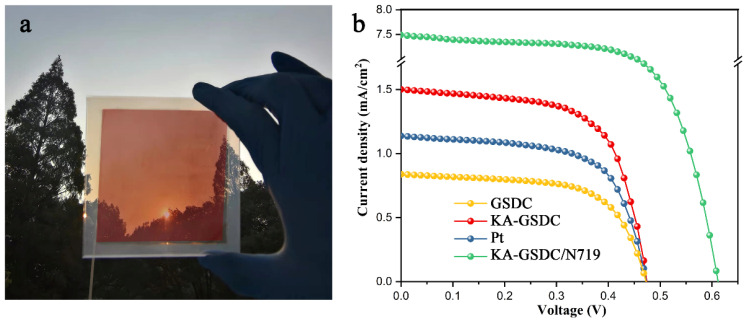
(**a**) The prepared DSSC setup; and (**b**) current–voltage characteristics of the fabricated DSSCs.

The grape skin extract-sensitized TiO_2_ film-coated FTO serves as the photoanode, which is integrated with the KA-GSDC/TiO_2_-coated FTO counter electrode and the iodine solution electrolyte to assemble a DSSC device (Figure 8a). The critical indicators for the performance of solar cells are presented in Table 2, including the photovoltaic efficiency (*η* (%)), the fill factor (*FF* (%)), the open-circuit voltage (*V*_OC_ (mV)), and the short circuit current density (*J*_SC_ (mA cm^−2^)), which are obtained from the J–V curves shown in Figure 8b. All three J–V curves are taken under the light intensity of 100 mW cm^−2^ at an air mass of AM 1.5 G (1 sun illumination) and a room temperature of 25 °C. The photovoltaic efficiency (*η* (%)) and fill factor (*FF* (%)) are calculated using the following equations [48]:

(8) $$\eta =((FF\times J_{\mathrm{SC}}\times V_{\mathrm{OC}})/P_{\mathrm{in}})\times 100\%$$

(9) $$FF=J_{m}V_{m}/J_{\mathrm{SC}}V_{\mathrm{OC}}$$

where *P*_in_ is the intensity of the incident light (W cm^−2^) and *J*_m_ (mA cm^−2^) and *V*_m_ (V) are the maximum current density and maximum voltage in the J–V curve, respectively, at the point of maximum power output.

Compared with the GSDC-based DSSC (0.27%), the DSSC device using the KA-GSDC as the counter electrode displays a higher photovoltaic efficiency of 0.48%, which is attributed to the improvement of the electrocatalytic activity of KA-GSDC due to its high surface area and hierarchical pore structure. The photovoltaic efficiency value is higher than some data for DSSCs using grape skin extract as photosensitizer, such as the wine grape-based DSSC (0.025%) [49], *Vitis labrusca* grape-based DSSC (0.061%) [50], and black grapes-based DSSC (0.43%) [51].

Moreover, a higher *J*_SC_ value of 1.52 mA cm^−^^2^ is achieved for the KA-GSDC-based device compared to that of the GSDC-based DSSC (0.85 mA cm^−2^). The faster the electrocatalysis of the electrode, the faster the redox reaction, which leads to the faster regeneration of dye molecules in the excited state in the I^−^/I^3−^ electrolyte [52]. The rapid regeneration of dye molecules improves the light-harvesting ability of photoanodes due to the production of plentiful photogenerated carriers, thus increasing the *J*_SC_ value. The improved *J*_SC_ is responsible for the higher photovoltaic efficiency of the fabricated KA-GSDC-based DSSC. In addition, the *η* of the KA-GSDC-based DSSC (0.48%) is 33% higher than that of the Pt-based DSSC (0.36%, Pt is a very common counter electrode material). Thus, the KA-GSDC can be regarded as a potential substitute for expensive Pt.

Open-circuit voltage is also an important parameter that determines the efficiency of DSSCs. The *V*_OC_ value of the KA-GSDC-based DSSC (0.48 V) is identical with that of the control group (i.e., the Pt-based DSSC). The open-circuit voltage is the difference between the Fermi level of the TiO_2_ electrode and the redox electrolyte potential, which mainly depends on the photosensitizer’s recombination rate and adsorption mode [53]. The existence of affluent alcohol groups in anthocyanins contributes to the strong binding of anthocyanins to the surface of TiO_2_ nanostructures by chemisorption (as confirmed by the blue shift phenomenon in Figure 3b). In addition, there are no electron donor groups and de-electron groups under the irradiation of light, which is the most crucial for the flow of electrons from dye molecules to TiO_2_. These characteristics ensure the high *V*_OC_ value of the KA-GSDC-based DSSC.

We employed KA-GSDC as a counter electrode and commercial dye (N719) as a photosensitizer to assemble a DSSC device. According to the experimental result, the open-circuit voltage (*V*_OC_) and the short circuit current density (*J*_SC_) values remarkably increase to 0.62 V and 7.63 mA cm^−2^, respectively, contributing to a high photovoltaic efficiency of 3.22%. The strong enhancement effect on photovoltaic properties, which originates from the replacement of photosensitizers (from natural dyes to commercial N719), has also been found in other studies [54,55]. The result further verifies the potential of KA-GSDC as an alternative to the Pt counter electrode. Moreover, the result indicates that it is necessary to modify the grape skin extract photosensitizer for further strengthening the photovoltaic property of DSSC devices.

Some recent reports of the DSSCs based on natural dyes are summarized in Table 3. In these studies, the precious metal Pt is used as the counter electrode, undoubtedly increasing the cost. In contrast, the photovoltaic efficiency of the grape skin extract-sensitized solar cell device using the inexpensive KA-GSDC as the counter electrode is comparable to or even higher than the data of these DSSCs based on other types of natural dyes and Pt counter electrode (0.05–0.47%).

### 3.5. EIS and IPCE of DSSC Devices

**Figure 9:**
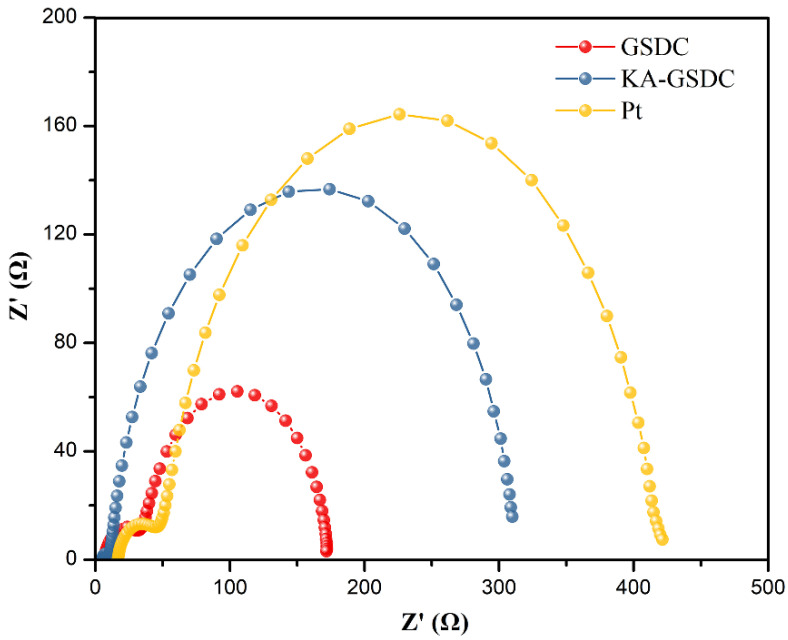
Nyquist plots of the fabricated GSDC-, KA-GSDC-, or Pt-based DSSC devices.

EIS has often been used to probe the kinetics and energetics of charge transport and recombination in DSSCs. EIS are recorded in the frequency range between 100 mHz and 100 kHz. Figure 9 displays the Nyquist plots of the fabricated GSDC-, KA-GSDC-, or Pt-based DSSC devices. The charge transfer resistance (*R*_1_) at the counter electrode–electrolyte interface and the charge recombination resistance (*R*_2_) at the TiO_2_–dye–electrolyte interface can be estimated from the EIS results. The diameters of the semicircles at the high-frequency and mid-frequency regions reflect the values of *R*_1_ and *R*_2_, respectively.

Based on the Nyquist plots in Figure 9, the *R*_1_ value of the KA-GSDC-based DSSC is lower than that of the GSDC- and Pt-based DSSCs. The lower *R*_1_ reflects the more efficient electron transfer between the electrocatalyst and the redox couples as a result of enhanced electrocatalysis. In addition, the *R*_2_ value of the KA-GSDC-based DSSC is higher than that of the GSDC-based DSSC and comparable with that of the Pt-based DSSC. The desired high value of *R*_2_ effectively hinders the recombination of electrolytic cations and photo-induced electrons in the conduction band of TiO_2_ [61]. The lower *R*_1_ and the higher *R*_2_ for the KA-GSDC-based DSSC are responsible for the highest photovoltaic efficiency observed among the three kinds of DSSC devices. In addition, the desired *R*_1_ and *R*_2_ values are mainly due to the enhanced electrocatalytic effect of the accessible, interconnected, and open pore structure of KA-GSDC, which contributes to more efficiently transferring electrons between the electrode and the electrolyte and enhancing the reduction reaction rate of redox couples [62].

IPCE is the ratio of generated electrons to incident photons, which relies on the light-harvesting efficiency and electron transfer yield that comprises the quantum charge injection and electron collection efficiency in the external circuit. Therefore, the higher is the IPCE, the higher the performance of a dye is, considering that all other factors are constant in the wake of change of the dyes. The IPCE value is calculated at respective excitation wavelength (*λ*) from the values of *J*_SC_ and the intensity of the corresponding monochromatic light (*P*_in_) using the following relation in Equation (10):

(10) $$\mathrm{IPCE}=\frac{1240\times J_{\mathrm{SC}}\left({\mathrm{mA}\;\mathrm{cm}}^{-2}\right)}{P_{\mathrm{in}}\left({\mathrm{mW}\;\mathrm{cm}}^{-2}\right)\times \lambda \left(\mathrm{nm}\right)}$$

**Figure 10:**
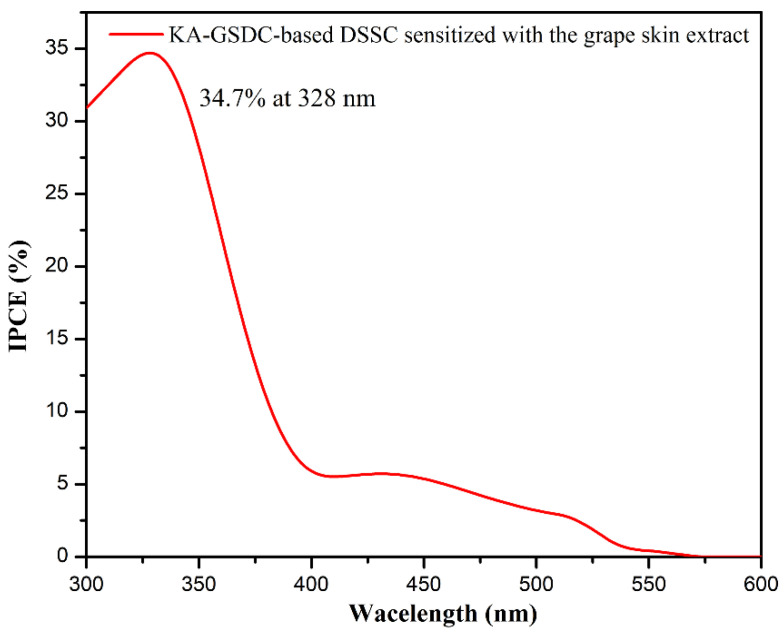
An action spectrum of IPCE for the KA-GSDC-based DSSC sensitized with the grape skin extract.

Figure 10 presents the IPCE spectrum of the KA-GSDC-based DSSC sensitized with the grape skin extract as a function of wavelength from 300 to 600 nm. As shown, the photoconversion of the DSSC mainly occurs in the ultraviolet and visible regions, and the device achieves a maximum IPCE value of 34.7% at 328 nm. Similarly, a distinct absorption can also be clearly identified in the UV–Vis absorption spectrum around the wavelength (Figure 3b). The IPCE value is higher than that of some similar nature dye-based DSSCs, such as the chlorophyll-based DSSC (6.1%) [63], betalain-based DSSC (9.9%) [63], and *Luffa cylindrica* L. extract-based DSSC (30%) [64]. The higher IPCE suggests an improved light scattering capacity and a high generation of charge carriers due to the better interaction between the photons and the dye molecules [65]. The improved generation of charge carriers results in the high *J*_SC_ value of KA-GSDC-based devices.

## 4. Conclusions

In summary, the dual application of waste grape skin is realized by using the grape skin and its extract as the carbon source of the counter electrode and the photosensitizer, respectively. The anthocyanins in the grape skin extract present strong binding with the TiO_2_ nanostructure on the photoanode, contributing to obtaining a high *V*_OC_ value of 0.48 V for the assembled DSSC device. The high surface area and hierarchical porous structure of KA-GSDC help to acquire an improved the electrocatalytic activity and a high *J*_SC_ value of 1.52 mA cm^−2^. Furthermore, the DSSC achieves a high photovoltaic efficiency of 0.48%, 33% higher than that of the Pt-based DSSC (0.36%). In addition, the efficiency is also comparable to or even higher than the data of these DSSCs based on other types of natural dyes and Pt counter electrode (0.05–0.47%). In addition, the DSSC achieves a maximum IPCE value of 34.7% at 328 nm. These results verify the potential of waste grape skin for the photosensitizer and counter electrodes in DSSCs.

## Figures and Tables

**Table 1:** ^1^H Chemical shifts used for metabolite identification (groups are indicated according to Fan et al. [38] and Pereira et al. [39]).

Compounds	Groups	*δ* ^1^H
Leucine	C5H_3_ + C6H_3_	0.92
Isoleucine	C5H_3_	1.01
Valine	C4H_3_ + C5H_3_	1.07
Arginine	C3H_2_	1.87
Proline	C4H_2_ + C3H_a_	2.07
Glutamine	C4H_2_	2.28–2.42
Citric acid	^1^/_2_(C2H_2_ + C4H_2_)	2.75
Malic acid	C2H_a_	2.62
	C2H_b_	2.85
Fructose	α C1H	3.66
-	β C1H	3.57
-	β C3H + β C4H	4.02
Glucose	β C2H	3.29
-	α C1H	4.55
-	β C1H	5.13
Sucrose	Glucopyranosyl-C1H	5.40
-	Glucopyranosyl-C2H	3.59
-	Glucopyranosyl-C3H	3.77
Phenolic compounds	-	5.74–7.37

**Table 2:** Solar cell parameters of the fabricated GSDC-, KA-GSDC-, or Pt-based DSSC devices.

Counter Electrode	*J*_SC_ (mA cm^−^^2^)	*V*_OC_ (V)	*FF*	*η* (%)
GSDC	0.85	0.49	0.65	0.27
KA-GSDC	1.52	0.48	0.65	0.48
Pt	1.15	0.48	0.65	0.36

**Table 3:** Solar cell parameters of some DSSCs based on natural dyes and the Pt counter electrode.

Dye	*J*_SC_ (mA cm^−^^2^)	*V*_OC_ (V)	*FF*	*η* (%)	Ref.
Rosella	1.63	0.40	0.57	0.37	[56]
Blue pea	0.37	0.37	0.33	0.05	[56]
Mixed rosella–blue pea	0.82	0.38	0.47	0.15	[56]
Bixin	1.10	0.57	0.59	0.37	[24]
Annatto	0.53	0.56	0.66	0.19	[24]
O. dillenii	1.09	0.52	0.69	0.47	[57]
T. indica	0.35	0.53	0.67	0.14	[57]
Dragon fruit	0.20	0.22	0.30	0.22	[58]
Spinach oleracea	1.11	0.58	0.46	0.29	[59]
Gardenia	1.29	0.56	0.48	0.35	[60]
Cochineal	0.51	0.78	0.25	0.10	[60]

## Data Availability

Data can be available upon request from the authors.
